# Prognostic Factors of Non-Predominant-Lepidic Lung Adenocarcinoma Presenting as Ground Glass Opacity: Results of a Multicenter Study

**DOI:** 10.3390/jpm14020153

**Published:** 2024-01-30

**Authors:** Fabiana Messa, Alessandra Siciliani, Giorgia Piccioni, Beatrice Leonardi, Anna Maria Ciccone, Antonio D’Andrilli, Claudio Andreetti, Cecilia Menna, Camilla Vanni, Alberto Emiliano Baccarini, Matteo Tiracorrendo, Massimiliano Mancini, Andrea Vecchione, Adriana Nocera, Giuseppe Calabrese, Elisa Meacci, Stefano Margaritora, Giovanni Natale, Alfonso Fiorelli, Federico Venuta, Erino Angelo Rendina, Giulio Maurizi, Mohsen Ibrahim

**Affiliations:** 1 Department of Thoracic Surgery, Sant’Andrea Hospital, Sapienza University, Via di Grottarossa 1035, 00189 Rome, Italy; messa.1665378@studenti.uniroma1.it (F.M.); alessandra.siciliani@uniroma1.it (A.S.); giorgia.piccioni@uniroma1.it (G.P.); annamaria.ciccone@ospedalesantandrea.it (A.M.C.); antonio.dandrilli@ospedalesantandrea.it (A.D.); claudio.andreetti@ospedalesantandrea.it (C.A.); cmenna@ospedalesantandrea.it (C.M.); alberto.baccarini@ospedalesantandrea.it (A.E.B.); matteo.tiracorrendo@uniroma1.it (M.T.); erinoangelo.rendina@uniroma1.it (E.A.R.); mohsen.ibrahim@uniroma1.it (M.I.); 2 Thoracic Surgery Unit, Università degli Studi della Campania “Luigi Vanvitelli”, 80131 Naples, Italy; beatrice.leonardi@unicampania.it (B.L.); giovanni.natale@unicampania.it (G.N.); alfonso.fiorelli@unicampania.it (A.F.); 3 Department of Clinical and Molecular Medicine, Sant’Andrea Hospital, 00189 Rome, Italy; mamancini@ospedalesantandrea.it (M.M.); andrea.vecchione@uniroma1.it (A.V.); 4 Division of Thoracic Surgery, Fondazione Policlinico Universitario A. Gemelli, IRCCS, 00168 Rome, Italy; adriana.nocera91@gmail.com (A.N.); giuseppe93calabrese@virgilio.it (G.C.); elisa.meacci@policlinicogemelli.it (E.M.); stefanomargaritore@unicatt.it (S.M.); 5 Division of Thoracic Surgery, Policlinico Umberto I, Sapienza University, 00161 Rome, Italy

**Keywords:** lung cancer, ground glass opacity, lepidic adenocarcinoma lymphadenectomy

## Abstract

This study aims to define the clinicopathological characteristics and prognosis of non-predominant lepidic invasive adenocarcinoma presenting as Ground Glass Opacity (GGO) nodules. The goal is to assess statistical relationships between histology, tumor size, location, and the incidence of relapse and lymph node dissemination. A retrospective multicenter study was conducted, including patients with GGO observed on CT scans between 2003 and 2021. Anamnestic, radiological, and histological data, as well as SUV values, lymphatic and vascular invasion, pathological stage, resection type, and adjuvant treatment, were analyzed. The primary endpoints were to evaluate prognostic factors for death and recurrence using Cox regression analysis. All 388 patients, including 277 with non-predominant lepidic invasive adenocarcinoma and 161 with lepidic adenocarcinoma, underwent curative anatomical resection. Non-predominant lepidic invasive adenocarcinoma demonstrated a worse prognosis than lepidic adenocarcinoma (*p* = 0.001). Independent prognostic factors for death and recurrence included lymph node involvement (*p* = 0.002) and vascular and lymphatic invasion (*p* < 0.001). In conclusion, non-predominant lepidic invasive adenocarcinoma and lymphatic and vascular invasion are prognostic factors for death and recurrence in GGO patients. Results suggest adjuvant treatment in the case of pN1-N2 disease, emphasizing the necessity of lymphadenectomy (sampling or systematic) for accurate staging and subsequent therapeutic procedures.

## 1. Introduction

**Figure 1:**
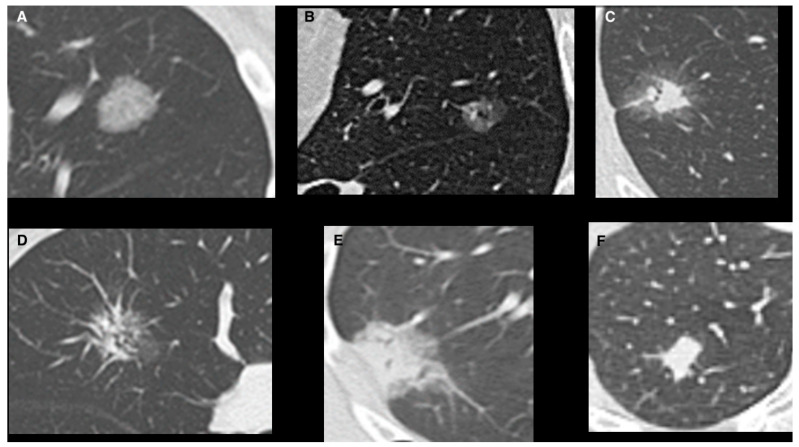
Suzuki classification of GGOs. (**A**) Homogeneous Pure GGO Type 1; (**B**) Homogeneous Semi-consolidation Type2; (**C**) Heterogeneous Halo Type 3; (**D**) Heterogeneous Mixed Type 4 (**E**) Solid/GGO Type 5; (**F**) Pure Solid Type 6.

**Figure 2:**
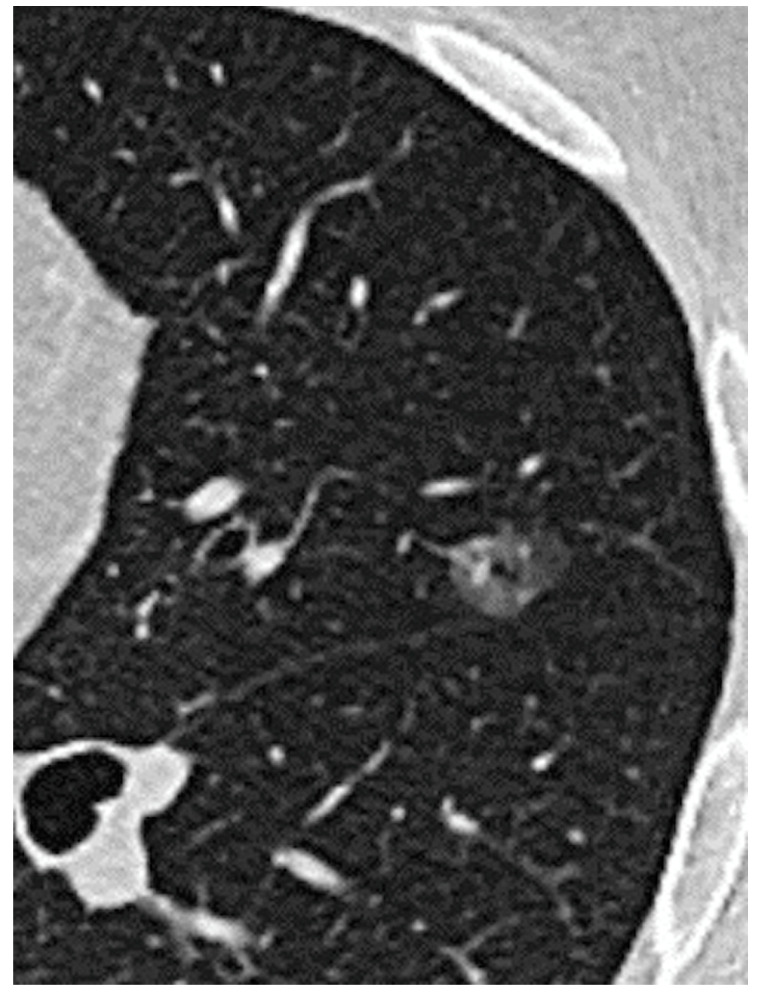
Ground Glass Opacity.

**Figure 3:**
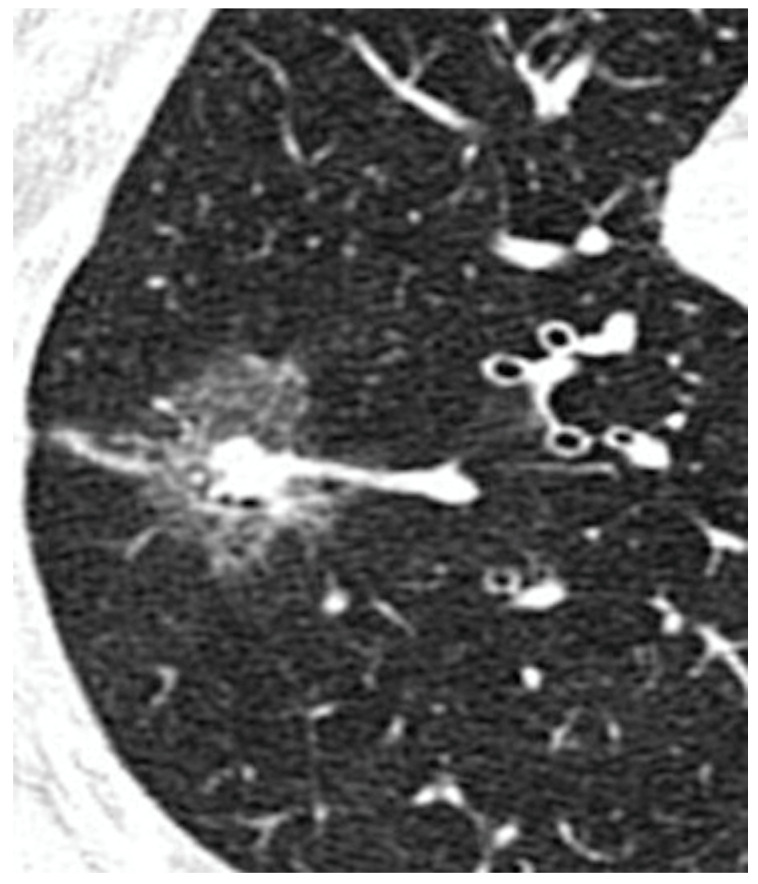
GGO’s Solid Component.

Lung adenocarcinoma is becoming the most frequent histological finding in lung cancer. According to the IASLC/ATS/ERS classification [1,2], adenocarcinoma is histologically classified as a preinvasive lesion, which includes atypical adenomatous hyperplasia and adenocarcinoma in situ characterized by a lepidic pattern, which is defined as the growth of neoplastic cells along alveolar septa without architectural destruction [2]. These lesions may progress to minimally invasive adenocarcinoma (MIA), characterized by an invasion less than 5 mm with a predominant lepidic pattern [3]. Invasive adenocarcinomas are classified by the histological component as lepidic, acinar, papillary, micropapillary, and solid [4]. Recently, imaging has proved to be useful in detecting some important clinical features and predicting histological characteristics of small ADC [5,6]. These lesions are therefore classified using the Suzuki classification (Figure 1): Homogeneous Pure GGO, Homogeneous semi-consolidation, Heterogeneous Halo (>50% GGO), Heterogeneous Mixed (>50% GGO), Solid GGO (<50% GGO) and Pure GGO [5,6]. Lepidic lesions often appear as pure Ground Glass Opacity (GGO) (Figure 2) or mixed GGO in relation to the radiologically solid component of the lesion [7,8] (Figure 3). Atypical adenomatous hyperplasia and adenocarcinoma in situ have excellent prognoses [9] with good survival, both with quite 100% disease-free survival at five years [10]. Although GGO-featured lung adenocarcinoma is commonly perceived as an indolent subtype, findings from natural history studies reveal that approximately 20% of pure GGOs and 40% of part-solid nodules demonstrate progression during follow-up. The progression dynamics vary, with some GGOs advancing rapidly while others remain stable for extended periods, spanning years or even decades. Despite these observations, the lack of an effective predictive method for GGO growth and the ongoing controversy surrounding the optimal timing for intervention persist as significant challenges. The correlation between the presence of ground glass opacities (GGOs) and favorable survival outcomes in lung adenocarcinoma is widely acknowledged. Moreover, Invasive adenocarcinomas with a predominantly lepidic component have a better prognosis than adenocarcinomas with other patterns [11,12]. Surgical resection is the first treatment option for GGO-featured lung adenocarcinoma.

The goal of this study was to evaluate any statistical relationship between the histological pattern, size, and location of invasive adenocarcinoma presenting as GGO on CT scan and the incidence of relapse, lymph node dissemination [13], and death outcome.

## 2. Materials and Methods

The study received approbation from the institutional review board (Prot. n. 7 SA/2023, RIF. CE 7031/2022). Written informed consent was obtained from all patients, and data were retrospectively analyzed. Between 2003 and 2021, 388 consecutive patients with a mixed or pure GGO observed on CT scan were included in a multicenter retrospective study, including Department of Thoracic Surgery, Sant’Andrea Hospital, Sapienza University of Rome; Thoracic Surgery Unit, Università degli studi della Campania “Luigi Vanvitelli” of Naples; Division of Thoracic Surgery, Fondazione Policlinico Universitario A. Gemelli, IRCCS, of Rome; and Division of Thoracic Surgery, Policlinico Umberto ISapienza University of Rome. All patients included in the study had histological confirmation of adenocarcinoma. No patient received a diagnosis of benignity. We analyzed anamnestic patient characteristics such as age, gender, comorbidities, and smoking status. Radiological and histological characteristics and SUV value were analyzed; lymphatic and vascular invasion, pathological stage, type of resection, and adjuvant treatment were statistically evaluated. All patients underwent lobectomy or sublobar resection with lymphadenectomy. All patients underwent Chest Computed Tomography (CT) with contrast before surgery. No patient underwent preoperative invasive hilar lymph node staging, considering that no suspicious lymphadenopathies were found on the preoperative CT. The majority of nodules considered for the study had a size of less than 3 cm. In the few cases where the size exceeded 3 cm, a preoperative PET/CT was performed, which did not show any uptake in the lymph nodes. For these reasons, preoperative lymph node staging was not conducted. Furthermore, when we refer to 3 cm, we are talking about the maximum extension, including the entire ground-glass opacity (GGO). The eighth edition of Lung Cancer Stage Classification suggests that the clinical T category be determined according to the invasive component: solid component size excluding the GGO component. As known in the literature, what influences prognosis, invasiveness, and lymph node involvement is the solid component of GGO, which is typically below 3 cm. None of the patients received neoadjuvant therapy before surgery. Patients enrolled in the study underwent pulmonary evaluation and function study using blood gas analysis by spirometry before surgery. Patients with cardiac disease were previously evaluated by a cardiologist. Each patient underwent lymph node dissection, sampling, or radical resection to perform correct pathological staging. All patients were analyzed based on their radiological and histological anamnestic characteristics. A total of 388 patients were divided into two groups based on histological characteristics. The two groups were, respectively, patients diagnosed with lepidic adenocarcinoma and patients diagnosed with non-predominant lepidic invasive adenocarcinoma. All the patients underwent mini-thoracotomy. The incidence of recurrence was investigated using radiologic and telephone follow-up. The last follow-up was in 2022.

### Statistical Analysis

Data were expressed as mean ± standard deviation (SD) for continuous variables and as absolute numbers and percentages for categorical variables. Data from the two groups were compared using the chi-square test for categorical variables and Student’s *t*-test for continuous variables. Cox regression analysis was performed to evaluate the prognostic factors for death and recurrence. Statistical significance was set at *p* < 0.05. MedCalc statistical software (version 12.3, Broekstraat 52; Mariakerke, Belgium) was used for the analysis.

## 3. Results

During the study period, 388 patients with ground-glass opacities who underwent curative surgical resection were enrolled. Two hundred twenty-seven patients (71%) were diagnosed with non-predominant lepidic invasive adenocarcinoma, and one hundred sixty-one (41%) were lepidic adenocarcinoma. Data from patients with non-predominant-lepidic (with acinar or papillary component) invasive adenocarcinoma were compared with those of patients with lepidic adenocarcinoma, as reported in Table 1. The rate of pure GGO was significantly higher in the patients with histology of lepidic adenocarcinoma than in those with non-predominant lepidic adenocarcinoma (67% vs. 24%, *p* < 0.0001). Two hundred fifty-five patients (66%) underwent lobectomy, 90 (23%) underwent segmentectomy, and forty-three (11%) underwent wedge resection. No significant differences were found in the site or side of the tumor location (*p* = 0.78), adjuvant chemotherapy (*p* = 0.86), or radiotherapy (*p* = 0.74). The mean tumor size was 1.96 ± 1.01, with no significant differences between the groups. Regarding lymph node involvement at pathological examination, 91% of the patients had N0, 7% had N1, and 2% had N2. Furthermore, patients with lymph node involvement who underwent adjuvant treatment experienced a better prognosis, with a reduction in recurrence (*p* < 0.002).

The recurrence rate was higher in the non-predominant lepidic adenocarcinoma group than in the lepidic adenocarcinoma group (19% vs. 8%, *p* = 0.003). The mean overall survival was significantly higher in the Lepidic adenocarcinoma group compared with the non-predominant-lepidic adenocarcinoma group (66.4 ± 38.9 months vs. 41 ± 31.4 months, *p* < 0.001). The recurrence and the death rate were higher in patients with lymph node involvement (*p* < 0.002 and *p* < 0.001, respectively). Also, disease-free survival was significantly higher in the lepidic adenocarcinoma group than in the non-predominant-lepidic adenocarcinoma group (64.9 ± 39.4 months vs. 37.7 ± 31 months, *p* < 0.001).

### Prognostic Factors for Death and Recurrence

Cox regression analysis showed that non-predominant lepidic invasive adenocarcinoma (*p* = 0.004), lymph node involvement (*p* = 0.001), pure GGO (*p* = 0.01), vascular invasion (*p* = 0.003), and lymphatic invasion (*p* = 0.001) were independent prognostic factors for death (Table 2). The analysis also showed that the independent prognostic factors for recurrence were lymph node involvement (*p* = 0.002), pure GGO (*p* = 0.003), non-predominant-lepidic invasive adenocarcinoma (*p* = 0.001), vascular invasion (*p* = 0.007), and lymphatic invasion (*p* = 0.002) (Table 3).

## 4. Discussion

In the era of low-dose CT scans and after the SARS-CoV2 pandemic, it is not unusual to detect ground glass opacity lesions as occasional findings. According to the radiological characteristics of GGO lesions, they have been defined as pure GGO or mixed GGO due to their subsolid or solid components [4,6]. The solid (or consolidation) component was defined as an area of increased opacification more than 5 mm in diameter, which completely obscured underlying vascular markings. Ground-glass opacity was defined as an area of a slight, homogeneous increase in density, which did not obscure underlying vascular markings. Semiconsolidation was defined as an area of an intermediate homogeneous increase in density that did not obscure underlying vascular markings. Mixed was an area with a heterogeneous increase in density, which consisted of GGO and a solid part with an air-bronchogram [6,14].

GGO lesions have been reported to have a good prognosis [15], and in most cases, their pathological features are minimally invasive [12]. According to Fu et al., the 5-year recurrence-free survival (RFS) rates for patients with pure GGO, part-solid, and solid nodules in invasive stage I non-small cell lung cancer (NSCLC) were documented at 100%, 87.6%, and 73.2%, respectively [15]. Many previous studies have focused on the radiological and histological correlations between pure GGO and the lepidic component or mixed GGO and the non-predominant-Lepidic component (with >60% of lepidic component) to identify subjects at risk of recurrence or lymph node involvement as soon as possible [5,6,12].

This study aimed to analyze the differences between two groups of patients based on their histopathological diagnosis: non-predominant-lepidic invasive adenocarcinoma and lepidic adenocarcinoma. The two groups were comparable in terms of anamnestic (medical history), radiological, and pathological characteristics. This is important to ensure that any observed differences in outcomes can be attributed to a specific histopathological diagnosis rather than to other variables. The Lepidic adenocarcinoma group had a higher correlation with radiologically pure GGOs. This suggests that the lepidic group may have a higher proportion of tumors with less invasive growth patterns. The recurrence rate was higher in the non-predominant-lepidic adenocarcinoma group than in the lepidic adenocarcinoma group. This finding implies that non-predominant lepidic adenocarcinomas are associated with a higher risk of recurrence after surgical resection. This demonstrates that pure GGOs are histologically characterized by predominant lepidic components. On the other hand, GGOs that show a solid or subsolid component on CT scans are more often associated with a non-predominant lepidic histological type with a worse prognosis [3,16,17,18,19]. The mean overall survival was significantly higher in the lepidic adenocarcinoma group than in the non-predominant-lepidic adenocarcinoma group. These findings suggest that besides the adenocarcinoma pathologic subtype, radiological features also play an important prognostic role that should not be neglected.

Additionally, disease-free survival was significantly higher in the lepidic adenocarcinoma group than in the non-predominant-lepidic adenocarcinoma group. This suggests that lepidic adenocarcinomas have a more favorable prognosis in terms of both overall and disease-free survival. These data are in line with the published experiences that include patients undergoing surgery for this peculiar lung cancer [12,20]. These findings have implications for the clinical management of patients with GGOs. Lepidic adenocarcinoma patients have better survival rates and lower recurrence rates than those presenting non-predominant lepidic adenocarcinoma.

The presence of certain radiological (such as mixed GGO) and pathological characteristics, such as lymph node involvement, vascular invasion, and lymphatic invasion, are associated with worse outcomes. This study confirmed, as other studies have already done, the importance of performing a correct lymphadenectomy in lepidic forms of adenocarcinoma, which, although less aggressive, can still result in recurrence and the need for adjuvant therapy. Plenty of studies [18] have demonstrated the superiority of radical lymph node dissection over lymph node sampling to guarantee better long-term survival and lower recurrence rates [10,12]. Many studies have confirmed that lymphadenectomy sampling can be considered optimal in patients with GGO [21,22].

In the context of pN2 disease, the data indicate a clear association between the presence of node involvement and an increased risk for recurrence and mortality. Therefore, the implication is that adjuvant treatment is suggested to reduce these elevated risks. The administration of treatment is envisioned as a strategic approach not only to reduce the likelihood of recurrence but also to decrease the overall death rate among patients. Conversely, for patients with pN1 involvement, the decision to recommend adjuvant oncological treatment may not be as straightforward. The results suggest that the necessity of such treatment may vary, and it might not be universally indicated for all patients with pN1 disease. This perspective reinforces the importance of precise staging through intraoperative lymphadenectomy, allowing for targeted therapeutic strategies that maximize benefits. In our center, we prefer to perform a systematic lymphadenectomy for a more accurate staging in those ground-glass opacities (GGO) that appear minimally invasive and, therefore, at risk of a worse prognosis with a higher recurrence rate. However, in other centers involved in this study, systematic lymphadenectomy was not always performed, opting for sampling instead. However, it would be interesting to extend the number of patients included in the study and to subject all patients to systematic lymphadenectomy in order to assess better pathological staging. Maurizi et al. suggested that performing a thorough nodal dissection can unveil nodal metastases in patients with lepidic adenocarcinoma, thereby enhancing the precision of pathologic staging. The presence of N1/N2 disease represents a negative prognostic factor, even within the context of this specific lung cancer histology. Consequently, a systematic lymphadenectomy should be duly considered in this setting to comprehensively address the potential impact on prognosis [12].

The present study presents some limitations. First, the limited sample of patients included, in fact, a larger number of patients, could validate the statistical results. The second limitation is the retrospective design, potentially introducing biases in data collection. Differences in adjuvant therapy and lymphadenectomy approaches across different centers may impact outcomes. The study acknowledges variations in lymphadenectomy practices in different centers, and this variability could represent a confounding factor.

In conclusion, this study highlights the possibility of categorizing lung adenocarcinoma according to radiological growth patterns. Moreover, our study focused on the correlation between pure GGO and the histological components of lepidic adenocarcinoma. Therefore, it underlines the importance of radiological characteristics in predicting prognosis and subsequent therapeutic procedures. Additionally, the study demonstrated that lesions radiologically presenting as solid or subsolid components (mixed GGOs) are often associated with a non-predominant histological pattern or adenocarcinoma. In the context of pN2 disease, the data indicate a clear association between the presence of lymph node involvement and an increased risk of recurrence and mortality. Lastly, some non-predominant lepidic adenocarcinoma patients included in this study showed N1/N2 disease. Therefore, lymphadenectomy should always be performed considering the worse prognosis in patients with N2 status.

## Figures and Tables

**Table 1:** Comparison between groups: Lepidic Adenocarcinoma and Non-Predominant-Lepidic Adenocarcinoma.

Variables	Total (*n* = 388)	Lepidic Adenocarcinoma (*n* = 161)	Non-Predominant-Lepidic Adenocarcinoma (*n* = 227)	*p*-Value
Age (years)	66.7 ± 7.25	65.9 ± 8.09	67.6 ± 6.27	0.19
History of cancer, *n* (%)	108 (27%)	46 (29%)	62 (27%)	0.78
Tumor size (cm)	1.96 ± 1.01	1.91 ± 0.95	1.99 ± 1.05	0.32
Pure GGO, *n* (%)	162 (42%)	108 (67%)	54 (24%)	<0.0001
N0, *n* (%)	353 (91%)	151 (94%)	202 (89%)	0.10
N1, *n* (%)	26 (7%)	8 (5%)	18 (8%)	0.25
N2, *n* (%)	9 (2%)	2 (1%)	7 (3%)	0.23
Right Upper Lobe, *n* (%)	123 (32%)	48 (30%)	75 (33%)	0.45
Right Middle Lobe, *n* (%)	18 (5%)	10 (6%)	8 (4%)	0.79
Right Lower Lobe, *n* (%)	71 (18%)	29 (18%)	42 (18%)	0.9
Left Upper Lobe, *n* (%)	117 (30%)	46 (29%)	71 (31%)	0.56
Left Lower Lobe, *n* (%)	59 (15%)	28 (17%)	31 (14%)	0.19
Segmentectomy, *n* (%)	90 (23%)	38 (24%)	52 (23%)	0.17
Lobectomy, *n* (%)	255 (66%)	111 (69%)	144 (63%)	0.26
Wedge resection, *n* (%)	43 (11%)	12 (7%)	31 (14%)	0.06
Adjuvant Chemotherapy, *n* (%)	66 (17%)	24 (15%)	42 (18%)	0.86
Radiotherapy, *n* (%)	16 (4%)	6 (4%)	10 (5%)	0.74
Recurrence, *n* (%)	58 (15%)	14 (8%)	44 (19%)	0.003
Disease Free Survival (months)	49.2 ± 37.3	64.9 ± 39.4	37.7 ± 31	<0.001
Overall Survival (months)	52.2 ± 36.8	66.4 ± 38.9	41 ± 31.4	<0.001

**Table 2:** Prognostic factors for death.

Variables	Hazard Ratio	95% Confidence Interval	*p*-Value
Age	2.3	0.78–3.4	0.32
Sex	3.4	0.34–2.9	0.23
Smoking status	4.3	0.78–3.4	0.45
SUV	1.4	0.59–4.7	0.56
Tumor location (central)	2.8	0.67–2.9	0.21
Tumor size	4.3	0.39–4.1	0.45
Lymph node involvement	3.4	2.3–5.6	0.001
Pure GGO	3.3	2.9–4.5	0.01
Non-lepidic adenocarcinoma	2.9	1.8–3.4	0.004
Lymphatic invasion	3.6	2.6–4.8	0.001
Vascular invasion	4.9	1.1–3.1	0.003

**Table 3:** Prognostic factors for recurrence.

Variables	Hazard Ratio	95% Confidence Interval	*p*-Value
Age	1.3	0.68–2.5	0.36
Sex	2.4	0.78–3.9	0.21
Smoking status	3.3	0.67–2.9	0.55
SUV	1.9	0.67–2.7	0.56
Tumor location (central)	2.4	0.56–3.8	0.31
Tumor size	3.7	0.56–3.8	0.55
Lymph node involvement	3.8	2.8–4.6	0.002
Pure GGO	2.3	2.8–4.8	0.003
Non-lepidic invasive carcinoma	3.9	1.8–4.4	0.001
Lymphatic invasion	4.6	3.1–4.1	0.002
Vascular invasion	4.2	1.8–3.9	0.007

## Data Availability

The original contributions presented in the study are included in the article, further inquiries can be directed to the corresponding author.
